# King’s stages of amyotrophic lateral sclerosis: an ^18^F-FDG-PET study of brain connectivity

**DOI:** 10.1093/brain/awag159

**Published:** 2026-05-08

**Authors:** Francesca Di Pede, Sara Cabras, Umberto Manera, Rosario Vasta, Grazia Zocco, Emilio Minerva, Enrico Matteoni, Filippo De Mattei, Giorgio Pellegrino, Francesca Palumbo, Daniela Pascariu, Stefano Callegaro, Alessandra Maccabeo, Giulia Polverari, Alessio Martino, Alessandro Giuliani, Cristina Moglia, Andrea Calvo, Adriano Chiò, Marco Pagani, Antonio Canosa

**Affiliations:** ALS Centre, ‘Rita Levi Montalcini’ Department of Neuroscience, University of Turin, Turin 10126, Italy; ALS Centre, ‘Rita Levi Montalcini’ Department of Neuroscience, University of Turin, Turin 10126, Italy; School of Advanced Studies, Centre for Neuroscience, University of Camerino, Camerino 62032, Italy; ALS Centre, ‘Rita Levi Montalcini’ Department of Neuroscience, University of Turin, Turin 10126, Italy; Neurology Unit 1U, Azienda Ospedaliero-Universitaria Città della Salute e della Scienza di Torino, Turin 10126, Italy; ALS Centre, ‘Rita Levi Montalcini’ Department of Neuroscience, University of Turin, Turin 10126, Italy; ALS Centre, ‘Rita Levi Montalcini’ Department of Neuroscience, University of Turin, Turin 10126, Italy; ALS Centre, ‘Rita Levi Montalcini’ Department of Neuroscience, University of Turin, Turin 10126, Italy; ALS Centre, ‘Rita Levi Montalcini’ Department of Neuroscience, University of Turin, Turin 10126, Italy; ALS Centre, ‘Rita Levi Montalcini’ Department of Neuroscience, University of Turin, Turin 10126, Italy; ALS Centre, ‘Rita Levi Montalcini’ Department of Neuroscience, University of Turin, Turin 10126, Italy; ALS Centre, ‘Rita Levi Montalcini’ Department of Neuroscience, University of Turin, Turin 10126, Italy; ALS Centre, ‘Rita Levi Montalcini’ Department of Neuroscience, University of Turin, Turin 10126, Italy; ALS Centre, ‘Rita Levi Montalcini’ Department of Neuroscience, University of Turin, Turin 10126, Italy; ALS Centre, ‘Rita Levi Montalcini’ Department of Neuroscience, University of Turin, Turin 10126, Italy; Positron Emission Tomography Centre AFFIDEA-IRMET S.p.A., Turin 10135, Italy; Department of AI, Data and Decision Sciences, LUISS Guido Carli University, Rome 00197, Italy; Environment and Health Department, Istituto Superiore di Sanità, Rome 00161, Italy; ALS Centre, ‘Rita Levi Montalcini’ Department of Neuroscience, University of Turin, Turin 10126, Italy; Neurology Unit 1U, Azienda Ospedaliero-Universitaria Città della Salute e della Scienza di Torino, Turin 10126, Italy; ALS Centre, ‘Rita Levi Montalcini’ Department of Neuroscience, University of Turin, Turin 10126, Italy; Neurology Unit 1U, Azienda Ospedaliero-Universitaria Città della Salute e della Scienza di Torino, Turin 10126, Italy; Neuroscience Institute of Turin (NIT), Turin 10124, Italy; ALS Centre, ‘Rita Levi Montalcini’ Department of Neuroscience, University of Turin, Turin 10126, Italy; Neurology Unit 1U, Azienda Ospedaliero-Universitaria Città della Salute e della Scienza di Torino, Turin 10126, Italy; Neuroscience Institute of Turin (NIT), Turin 10124, Italy; Institute of Cognitive Sciences and Technologies, C.N.R., Rome 00196, Italy; Institute of Cognitive Sciences and Technologies, C.N.R., Rome 00196, Italy; Department of Medical Radiation Physics and Nuclear Medicine, Karolinska University Hospital, Stockholm 17177, Sweden; ALS Centre, ‘Rita Levi Montalcini’ Department of Neuroscience, University of Turin, Turin 10126, Italy; Neurology Unit 1U, Azienda Ospedaliero-Universitaria Città della Salute e della Scienza di Torino, Turin 10126, Italy; Neuroscience Institute of Turin (NIT), Turin 10124, Italy; Institute of Cognitive Sciences and Technologies, C.N.R., Rome 00196, Italy

**Keywords:** amyotrophic lateral sclerosis, King’s staging system, ^18^F-FDG-PET, connectivity

## Abstract

Amyotrophic lateral sclerosis (ALS) is a fatal neurodegenerative disease affecting upper and lower motor neurons. TAR DNA-binding protein 43 (TDP-43) proteinopathy is the neuropathological signature of the disease, and ^18^F-fluorodeoxyglucose PET (^18^F-FDG-PET) serves as a marker of neurodegeneration *in vivo*. The aim of the present cross-sectional study was to disentangle ^18^F-FDG-PET correlates of disease severity assessed through the King’s staging system, by exploring connectivity changes across motor stages.

Patients with ALS classified as King’s stage 1, 2 or 3, who underwent brain ^18^F-FDG-PET at diagnosis from 2008 to 2022 at the ALS Centre of Turin, were included. A multiple regression analysis to evaluate the relationship between brain metabolism and King’s stage was performed. The clusters showing significant results were used as seed regions in an interregional correlation analysis (IRCA), performed for each stage.

Of a total of 832 patients with ALS, 337 were classified as King’s stage 1, 274 as stage 2 and 221 as stage 3. The three groups significantly differed in age at PET, disease duration and total ALS Functional Rating Scale Revised (ALSFRS-R) score at the time of PET, *C9orf72* status and the distribution of cognitive categories. We found a decreasing metabolic gradient from King’s stage 1 to King’s stage 3 in a cluster encompassing motor and cognitive areas. As King’s stage increases, we found a decrease of connectivity within the sensorimotor and cognitive areas. The IRCA also showed the connectivity of motor and cognitive regions with temporal and cerebellar regions. The connectivity with temporal regions found in King’s stage 1 decreases in King’s stage 2 and finally, disappears in King’s stage 3. The connectivity with the cerebellum occurs in King’s stage 2 and decreases in King’s stage 3.

The changes of connectivity of motor and cognitive areas with temporal and cerebellar regions among different King’s stages might reflect the spread of TDP-43 proteinopathy or a compensatory mechanism, respectively. The present study suggests that ^18^F-FDG-PET imaging of the brain may be integrated with the King’s staging system to assess the extent of the pathogenic process in the context of clinical trials.

## Teaser

Di Pede *et al.* show that advancing ALS motor stages are associated with reduced metabolic connectivity within sensorimotor and cognitive areas, with stage-specific changes involving temporal and cerebellar regions. ^18^F-FDG-PET may complement King’s staging to assess neurodegeneration in clinical trials.

**See Dubbioso and Pappatà (https://doi.org/10.1093/brain/awag211) for a scientific commentary on this article.**

## Introduction

Amyotrophic lateral sclerosis (ALS) is a neurodegenerative disease affecting upper and lower motor neurons and leading to progressive motor disability until death, which usually occurs because of respiratory failure 3–5 years after disease onset.^1^ TAR DNA-binding protein 43 (TDP-43)-positive intracytoplasmic aggregates in neurons are the neuropathological signature of the disease.^2^ TDP-43 proteinopathy is thought to spread from the motor cortex to anterior and posterior brain regions, possibly with a prion-like behaviour, via axonal connections.^3,4^ The King’s clinical staging system classifies patients with ALS primarily based on the anatomical extent of the disease. Stages 1, 2 and 3 correspond to the involvement of one, two or three body regions respectively, while stage 4 is reached when gastrostomy or non-invasive ventilation (NIV) are required (defining stages 4A and 4B, respectively). Supporting the validity of the model, King’s stages progress in distinct temporal intervals and have an inverse correlation with survival time.^5^ Additionally, staging can be assigned retrospectively using the ALS Functional Rating Scale Revised (ALSFRS-R)-based algorithm proposed by Balendra *et al*.^6^ In a previous study from our centre, we found clusters of relative hypometabolism in advanced clinical disease stage (King’s stage 3) compared with earlier clinical stages (King’s stages 1 and 2). These clusters included the motor and premotor cortex, extending towards anterior regions, hence mirroring TDP-43 spreading according to neuropathological stages.^7^ The aim of the present study is to further disentangle, by ^18^F-FDG-PET, the brain metabolic correlates of disease severity assessed through the King’s staging system, by exploring not only brain metabolism *per se* but also connectivity changes across motor stages, to elucidate the underlying pathophysiological mechanisms.

## Materials and methods

### Participants

We considered eligible for the study all patients diagnosed with definite, probable and probable laboratory-supported ALS according to El Escorial revised diagnostic criteria^8^ and classified at diagnosis as King’s stages 1, 2 or 3 according to the ALSFRS-R-derived algorithm by Balendra *et al*.,^6^ who underwent brain ^18^F-FDG-PET at diagnosis between 2008 and 2022 at the ALS Centre of Turin (‘Rita Levi Montalcini’ Department of Neuroscience, University of Turin, Turin, Italy). Stage 4 was not included as it reflects functional milestones (i.e. NIV or gastrostomy) rather than the anatomical extent of motor impairment. Patients with major systemic illnesses, major vision disturbances, psychiatric illnesses, drug and/or alcohol addiction, and other diseases potentially affecting brain functioning and metabolism were also excluded.

Patients underwent a series of neuropsychological tests assessing executive function, verbal and visual memory, attention and working memory, visuo-spatial function, language, social cognition and behaviour. The tests were administered at diagnosis. The tests were selected according to the diagnostic criteria for behavioural variant frontotemporal dementia (bvFTD)^9^ and the amyotrophic lateral sclerosis-frontotemporal dementia (ALS-FTD) consensus criteria.^10^ A list of the tests, along with their classification according to the main neuropsychological domain, can be found in the Supplementary material, Methods. Based on the full neuropsychological assessment, patients were classified according to the ALS-FTD consensus criteria as: patients with normal cognition (ALS-CN); patients with isolated cognitive impairment (ALSci); patients with isolated behavioural impairment (ALSbi); patients with both cognitive and behavioural impairment (ALScbi); and patients with frontotemporal dementia (ALS-FTD).

### ^18^F-FDG-PET scan acquisition and preprocessing

Brain ^18^F-FDG-PET was performed according to published guidelines.^11^ Patients fasted at least 6 h before the exam. Blood glucose was <7.2 mmol/l in all cases before the procedure. After a 20-min rest, about 185 MBq of ^18^F-FDG was injected. The acquisition started 60 min after the injection. PET/CT scans were performed on a Discovery ST-E System (General Electric) and on a Discovery IQ System (General Electric). Brain CT and PET scans were sequentially acquired, the former being used for attenuation correction of PET data. The PET images were reconstructed with four iterations and 28 subsets with an initial voxel size of 2.34 × 2.34 × 2.00 mm and data were collected in 128 × 128 matrices. Images were spatially normalized to a customized brain ^18^F-FDG-PET template^12^ and subsequently smoothed with a 10-mm filter in MATLAB R2018b (MathWorks). Intensity normalization at individual level averaging each voxel for the mean value of the whole brain was performed.

### Statistical analyses

To assess the differences among the three groups (King’s 1, 2 and 3) regarding the main clinical and demographic characteristics, the following tests were employed: the Chi-square test for nominal categorical variables; the non-parametric Kruskal–Wallis test for continuous quantitative variables with significant Levene test and Shapiro–Wilk test results, where the assumptions of homogeneity of variances and normality of distribution required for ANOVA were not met.

The relationship between King’s stage (1, 2, 3) and relative brain metabolism was investigated using the multiple regression model of SPM12. The covariates have been included in the analysis according to the results of the descriptive statistics of demographic and clinical data of the sample reported later. The clusters showing significant correlations were then extracted and used as seed regions to perform a voxel-wise inter-regional correlation analysis (IRCA), regressing the metabolism of the seed regions against that of the whole brain for each of the three groups (King’s stages 1, 2 and 3) in order to identify eventual connectivity differences. In all the analyses, only clusters containing ≥100 contiguous voxels were considered significant. Brodmann areas (BAs) were identified at a 0–2 mm range from the Talairach coordinates of the SPM output isocentres corrected by Talairach Client (http://www.talairach.org/index.html).

### Ethical standards

The study was approved by the ethical committee ‘Comitato Etico Interaziendale Azienda Ospedaliero-Universitaria Città della Salute e della Scienza di Torino.’ The study was performed in accordance with the ethical standards as laid down in the 1964 Declaration of Helsinki and its later amendments or comparable ethical standards. Participants signed a written informed consent. They did not receive any remuneration for participation. Data were anonymized according to the European regulations for the protection of privacy.

## Results

### Clinical and demographic data

Overall, *n* = 832 patients were included in the study. This reflects a participation rate of 31.14% among patients from the Piemonte and Valle d’Aosta Registry for ALS (PARALS) during the study period. Conversely, a precise estimate cannot be calculated for subjects coming from other Italian regions and thus are not included in this registry. Based on the ALSFRS-R at the time of PET, 337 patients were classified as King’s stage 1, 274 as stage 2 and 221 as stage 3. The demographic and clinical characteristics of the three groups and the overall sample are detailed in Table 1. Most PET scans were performed using the Discovery ST-E System (GE) scanner. However, since a minority were acquired with the Discovery IQ System (GE), we included the type of scanner among the covariates in the analysis.

**Table 1:** Clinical and demographic characteristics of the whole sample and of the three groups separated (King’s stages 1–3)

		King’s stage			*P*-value (test statistics)	Total (*n* = 832)
		1 (*n* = 337)	2 (*n* = 274)	3 (*n* = 221)		
Sex	Female, *n* (%)	141 (41.8%)	114 (41.6%)	106 (48.0%)	*P* = 0.277	361 (43.4%)
	Male, *n* (%)	196 (58.2%)	160 (58.4%)	115 (52.0%)	(χ^2^ = 2.57)	471 (56.6%)
	Total, *n*	337	274	221	–	832
Age at PET, years, median (IQR)		63.0 (56.0–70.0)	63.0 (54.0–71.0)	67.0 (57.0–72.0)	***P* = 0.006** (χ^2^ = 10.3)	64.0 (56.0–71.0)
Disease duration at PET, months, median (IQR)		11.7 (8.27–16.8)	12.2 (8.72–17.7)	13.3 (8.83–21.2)	***P* = 0.018** (χ^2^ = 8.06)	12.3 (8.53–18.0)
Onset	Bulbar, *n* (%)	103 (30.6%)	67 (24.5%)	75 (33.9%)	*P* = 0.078	245 (29.4%)
	Spinal, *n* (%)	231 (68.5%)	207 (75.5%)	145 (65.6%)	(χ^2^ = 8.38)	583 (70.1%)
	FTD, *n* (%)	3 (0.9%)	0 (0.0%)	1 (0.5%)	–	4 (0.5%)
	Total, *n*	337	274	221	–	832
*C9orf72*	No expansion, *n* (%)	293 (90.2%)	245 (91.4%)	205 (96.2%)	***P* = 0.031**	743 (92.2%)
	Expansion, *n* (%)	32 (9.8%)	23 (8.6%)	8 (3.8%)	(χ^2^ = 6.95)	63 (7.8%)
	Total, *n*	325	268	213	–	806
ALSFRS-R Total, median (IQR)		44.0 (42.0–45.0)	40.0 (37.0–42.0)	34.0 (30.0–38.0)	***P* < 0.001** (χ^2^ = 434)	40.0 (36.0–43.0)
Strong category	ALS-cn, *n* (%)	182 (67.9%)	126 (60.3%)	86 (50.9%)	***P* = 0.003**	394 (61.0%)
	ALS-bi, *n* (%)	17 (6.3%)	27 (12.9%)	25 (14.8%)	(χ^2^ = 23.5)	69 (10.7%)
	ALS-ci, *n* (%)	46 (17.2%)	34 (16.3%)	26 (15.4%)	–	106 (16.4%)
	ALS-cbi, *n* (%)	11 (4.1%)	11 (5.3%)	17 (10.1%)	–	39 (6.0%)
	ALS-FTD, *n* (%)	12 (4.5%)	11 (5.3%)	15 (8.9%)	–	38 (5.9%)
	Total, *n*	268	209	169	–	646
PET scanner	Discovery ST–E System (GE), *n* (%)	304 (90.2%)	254 (92.7%)	202 (91.4%)	*P* = 0.552 (χ^2^ = 1.19)	760 (91.3%)
	Discovery IQ System (GE), *n* (%)	33 (9.8%)	20 (7.3%)	19 (8.6%)	–	72 (8.7%)
	Total, *n*	337	274	221	–	832

Statistically significant *P*-values are highlighted in bold. ALS = amyotrophic lateral sclerosis; ALSFRS-R = ALS Functional Rating Scale; ALS-cn = ALS with normal cognition; ALS-bi = ALS with behavioural impairment; ALS-ci = ALS with cognitive impairment; ALS-cbi = ALS with cognitive and behavioural impairment; ALS-FTD = ALS with frontotemporal dementia; IQR = interquartile range.

The three groups (King’s stages 1, 2 and 3) significantly differed in the following variables: age at PET, disease duration and total ALSFRS-R score at the time of PET, *C9orf72* status and the distribution of cognitive categories according to Strong classification defined by revised 2017 criteria.^10^ Therefore, age at PET was included as a covariate in the analysis assessing the correlation between brain metabolism and King’s stage. However, we decided not to adjust for the other variables that showed significant differences for the following reasons. The observed differences among the three groups in terms of total ALSFRS-R score and disease duration are expected, as King’s stage is derived from the ALSFRS-R using the algorithm proposed by Balendra *et al*.,^6^ and it is reasonable to assume that a more advanced stage at the time of diagnosis might be more likely associated with a longer disease duration. Finally, the different distribution of cognitive impairment across King’s stages has already been reported in the published literature,^13^ suggesting that cognitive and motor decline might occur in parallel. Given that cognitive and behavioural impairment are now widely regarded as an integral aspect of the disease, and since the objective of this study was to analyse the metabolic and connectivity changes associated with the varying degrees of disease severity in its overall context, it was decided that cognitive category should not be included as a covariate in subsequent PET analyses. Although we did not observe any difference in female/male ratio among the three groups, we included sex as a covariate in the analyses, based on our previous study pointing out the impact of sex on brain metabolism in ALS.^14^ Nevertheless, to avoid misinterpretation of our results, we also ran separate sensitivity analyses including *C9orf72* status, site of onset (spinal versus bulbar), disease duration at PET and cognitive status at PET (i.e. cognitive category according to ALS-FTD consensus criteria) as covariates.

### ^18^F-FDG-PET data

#### Correlation between brain metabolism and King’s stage

**Figure 1:**
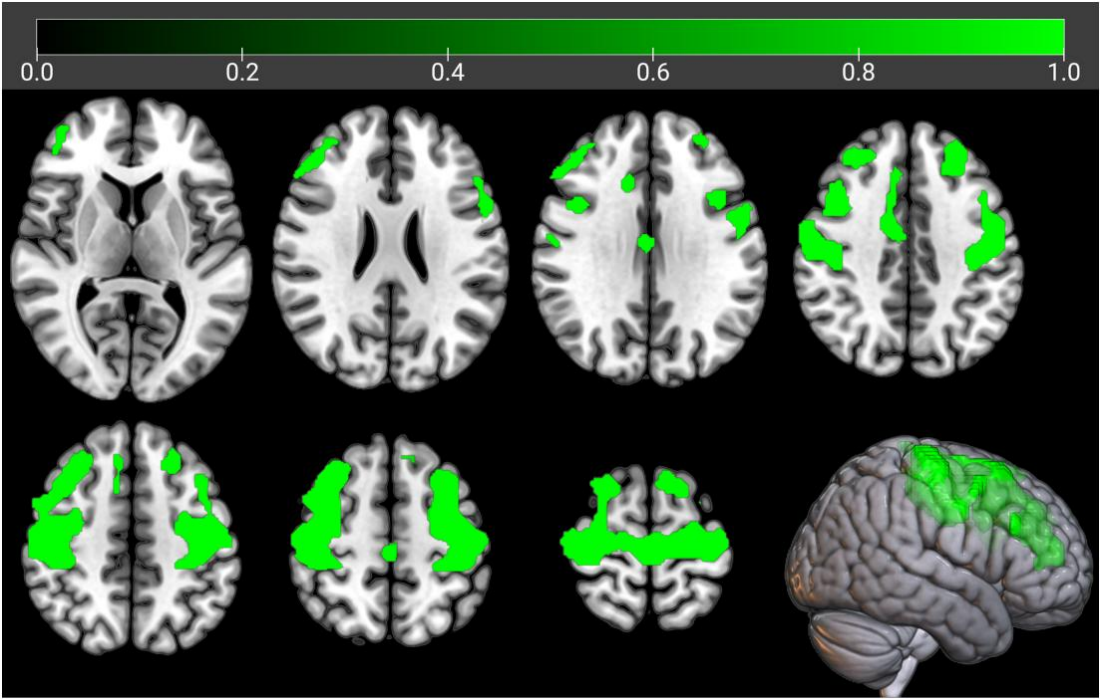
**Correlation between brain metabolism and King’s stage.** Clusters of significant negative correlation between brain metabolism and King’s stage are marked in green and are reported on axial sections of a brain MRI template and on the brain surface of a glass brain rendering (*bottom right*).

At the height threshold of *P* < 0.001 [family wise error (FWE)-corrected for multiple comparisons at cluster level], a negative correlation was observed in a cluster including the bilateral precentral gyri, bilateral postcentral gyri, right middle frontal gyrus, bilateral superior frontal gyri, left inferior frontal gyrus, right anterior cingulate gyrus and right medial frontal gyrus (Fig. 1 and Supplementary Table 1).

No cluster of positive correlation between brain metabolism and King’s stages was found. The scatter plot of negative correlation between brain metabolism and King’s stage is reported in Supplementary Fig. 1.

The sensitivity analyses for *C9orf72* status (*n* = 806 subjects), site of onset (*n* = 832) and disease duration at PET (*n* = 830) did not yield different results (Supplementary Fig. 2). The sensitivity analysis including cognitive status as a covariate confirmed the inverse relationship between King’s stage and brain metabolism in motor regions, while the cognitive regions were no longer detectable in the cluster (Supplementary Fig. 2).

#### Interregional correlation analysis

The height threshold was set at *P* < 0.00001 (FWE-corrected for multiple comparisons) in all runs of the IRCA. Based on the very large sample, we adopted this very stringent threshold to be more conservative and cautious in interpreting the results.

##### King’s stage 1: positive correlation

**Figure 2:**
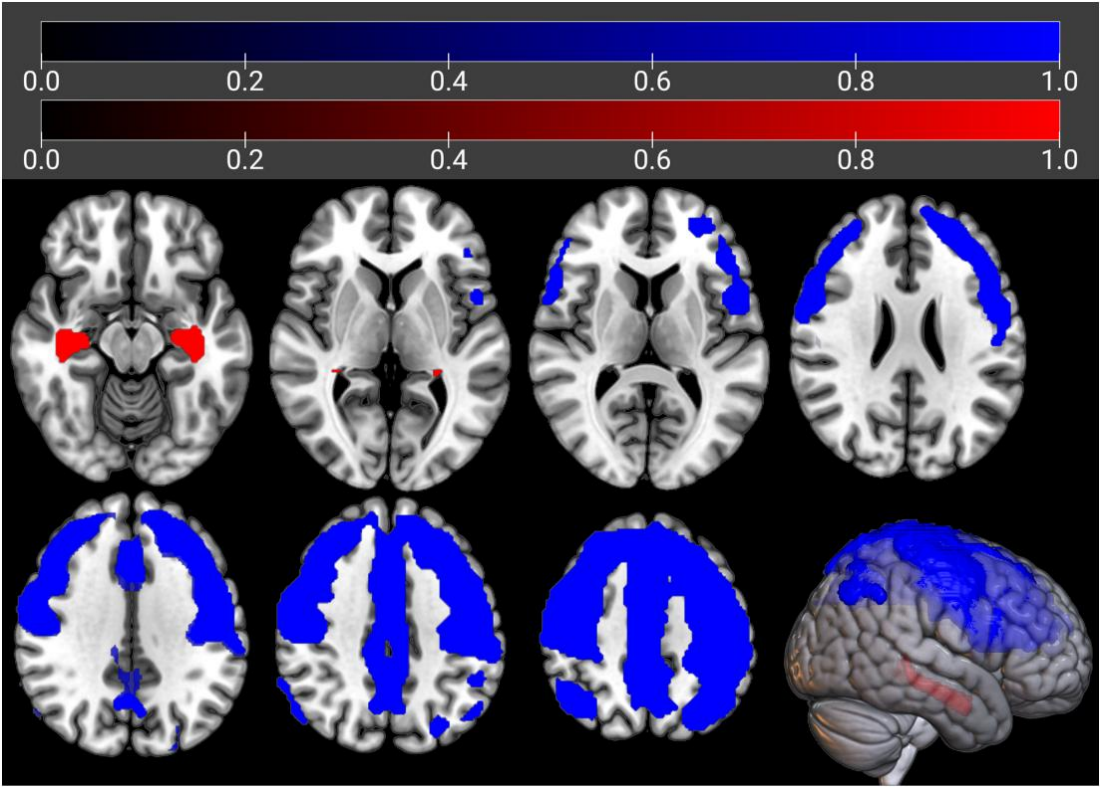
**IRCA in King’s 1 patients.** Clusters of significant positive (blue) and negative (red) interregional correlation analysis (IRCA) between the metabolism of the seed region and the metabolism of the whole brain in King’s stage 1 are reported on axial sections of a brain MRI template and on the brain surface of a glass brain rendering (*bottom right*).

In the King’s stage 1 group, a positive correlation of metabolism was found between the seed region and a cluster including the bilateral middle frontal gyri, bilateral precentral gyri, left postcentral gyrus, bilateral medial frontal gyri, right superior frontal gyrus and left paracentral lobule (Fig. 2 and Supplementary Table 2).

##### King’s stage 1: negative correlation

In the King’s stage 1 group, a negative correlation of metabolism was found between the seed region and a cluster including the bilateral medial temporal regions, bilateral caudate tail and right parahippocampal gyrus (Fig. 2 and Supplementary Table 3).

##### King’s stage 2: positive correlation

**Figure 3:**
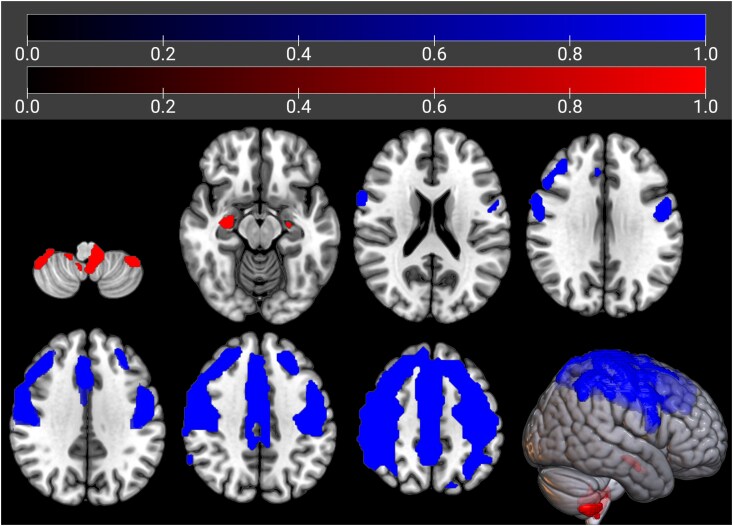
**IRCA in King’s 2 patients.** Clusters of significant positive (blue) and negative (red) interregional correlation analysis (IRCA) between the metabolism of the seed region and the metabolism of the whole brain in King’s stage 2 are reported on axial sections of a brain MRI template and on the brain surface of a glass brain rendering (*bottom right*).

In the King’s stage 2 group, a positive correlation of metabolism was found between the seed region and a cluster including the bilateral precentral gyri, bilateral middle and medial frontal gyri, right superior frontal gyrus and right inferior parietal lobule (Fig. 3 and Supplementary Table 4).

##### King’s stage 2: negative correlation

In the King’s stage 2 group, a negative correlation of metabolism was found between the seed region and a cluster including the left cerebellar tonsil (and surrounding bilateral cerebellar white matter), bilateral parahippocampal gyri (right amygdala and left hippocampus), bilateral sub-gyral temporal regions (right BA 20 and left BA 21) and left fusiform gyrus (Fig. 3 and Supplementary Table 5).

##### King’s stage 3: positive correlation

**Figure 4:**
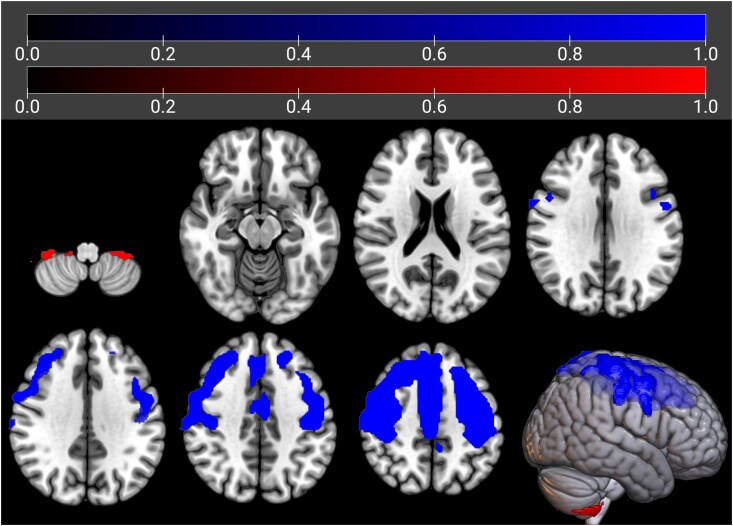
**IRCA in King’s 3 patients.** Clusters of significant positive (blue) and negative (red) interregional correlation analysis (IRCA) between the metabolism of the seed region and the metabolism of the whole brain in King’s stage 3 are reported on axial sections of a brain MRI template and on the brain surface of a glass brain rendering (*bottom right*).

In the King’s stage 3 group, a positive correlation of metabolism was found between the seed region and a cluster including the bilateral precentral gyri, bilateral middle frontal gyri, bilateral medial frontal gyri, bilateral superior frontal gyri, left anterior cingulate gyrus and left postcentral gyrus (Fig. 4 and Supplementary Table 6).

##### King’s stage 3: negative correlation

In the King’s stage 3 group, a negative correlation of metabolism was found between the seed region and a cluster involving the bilateral cerebellum (left inferior semi-lunar lobule, right cerebellar tonsil and surrounding bilateral cerebellar white matter) (Fig. 4 and Supplementary Table 7).

## Discussion

The aim of this study was to explore connectivity changes across King’s stages in ALS. We focused on King’s stages 1, 2 and 3 because they correspond to the involvement of one, two or three body regions, respectively.^5,6^ King’s stage 4 was not considered because it represents functional milestones (i.e. need for gastrostomy or NIV), rather than a higher anatomical extent of the disease. The participation rate appears appropriate given the inherent challenges of conducting neuroimaging studies in ALS, particularly in the context of rapidly progressive motor disability. Furthermore, the use of highly stringent statistical thresholds, combined with the large sample size, enhances the robustness of our findings. The large cohort included in this study also enabled us to assess the impact on our findings of key demographic and clinical variables relevant to characterizing the disease phenotype and known to influence cerebral metabolism. We did not include a healthy control group since the point of the comparison between different King’s stage groups and healthy controls was already addressed in a previous publication from our centre.^7^ In that study the comparisons between each stage group and healthy controls showed the extension of frontal relative hypometabolism from stage 1 to stage 3, with a larger metabolic gap between stages 2 and 3.

In the multiple regression analysis, we found a decreasing metabolic gradient from King’s stage 1 to King’s stage 3 in a cluster including the bilateral precentral gyri (BA 4 and 6), bilateral postcentral gyri (left BA 4 and right BA 3), right middle frontal gyrus (BA 6 and 9), bilateral superior frontal gyri (BA 8), left inferior frontal gyrus (BA 45), right cingulate gyrus (BA 24 and 32) and right medial frontal gyrus (BA 8). This cluster encompasses motor and cognitive areas. Indeed, BA 4, 6 and 3 correspond to the primary motor, premotor and part of primary somatosensory cortices, respectively, which are known to provide a major contribution to the origin of the corticospinal tracts. Instead, BA 8, 9 and 45 belong to the prefrontal cortex, processing cognitive and behavioural functions. Moreover, a previous study found clusters of relative hypometabolism also including BA 8, 9, 45, 24 and 32 when comparing ALS patients showing different degrees of severity of cognitive and behavioural impairment with cognitively normal patients with ALS.^15^ Finally, synaptic degeneration in BA 9 has been identified as a predictor of cognitive impairment in ALS.^16^ Our sensitivity analyses showed that *C9orf72* status, site of onset and disease duration at PET did not have any impact on the results. After the inclusion of cognitive status as a covariate, the inverse relationship between King’s stage and brain metabolism was still evident in motor regions whereas in cognitive regions it was no longer detectable. This finding was expected and confirms that cognitive status is associated with relatively lower metabolism of those extra-motor regions. The findings of the present study expand our previous results obtained in a subset of the present cohort^7^ and provide further confirmation to the hypothesis that motor and cognitive impairment follow a parallel pattern of severity.^13^ Based on our data and in agreement with the ALS-FTD continuum hypothesis, motor stage and cognitive status seem to be two interrelated components of the same neurodegenerative process.

The true novelty of this study comes from the assessment of brain connectivity. The metabolism of the seed region has been shown to decrease as King’s stage increases in the first step of the analysis, in agreement with neuropathological data on disease staging.^4^ When IRCA was applied, we found a large autocorrelation with the seed region across the three stages. Although this may appear redundant, we can appreciate a progressive reduction in the extent of the clusters as King’s stage increases. This observation suggests a gradual decline in connectivity within sensorimotor and cognitive areas as progression occurs from King’s stage 1 to King’s stage 3. This is in line with a recent MRI study demonstrating a progressive decrease of intra-network and inter-network structural connectivity of sensorimotor regions as ALS stage increases.^17^

In contrast, the metabolism of the seed region demonstrated a negative correlation with the clusters including the bilateral temporal regions and bilateral caudate tails in King’s stage 1, bilateral temporal regions and right cerebellar tonsil in King’s stage 2, and bilateral cerebellar regions in King’s stage 3. Namely, as metabolism decreases in the seed region, metabolism initially increases in the temporal lobe in King’s stages 1 and 2, and subsequently in the cerebellum in King’s stages 2 and 3. Specifically, the extent of the temporal cluster and the cerebellar cluster (and consequently the connectivity in those regions) decreases when progressing from King’s stage 1 to King’s stage 2 and from King’s stage 2 to King’s stage 3, respectively. The interpretation of the negative IRCA is challenging since connectivity could represent either a compensatory mechanism or a facilitating path for TDP-43 spreading. It has been suggested that increased connectivity might be protective when it occurs towards metabolically preserved regions, while it might be maladaptive when it occurs towards affected regions.^18^ Moreover, hyperconnectivity in regions that play a central role in neural networks (i.e. hubs) may reflect an adaptive mechanism in the early stages, which is progressively exhausted by excessive functional load over time.^19^ Despite the general consensus that it remains impossible to distinguish between positive and negative effects of hyperconnectivity in the absence of longitudinal data with sufficient sample sizes,^20^ the inclusion of clinical stage as a surrogate for disease progression, as shown in the present study, may serve this purpose. According to neuropathological stages by Brettschneider *et al*.,^4^ TDP-43 proteinopathy affects the basal ganglia (i.e. striatum, including caudate nucleus) in stage 3 and anteromedial areas of the temporal lobe and the hippocampal formation in stage 4. Apart from a minority of stage 4 cases which displayed mild involvement of the dentate nucleus, the cerebellum was generally spared. Therefore, we could speculate that our findings regarding the temporal and the cerebellar clusters underpin different pathophysiological phenomena. The connectivity towards temporal regions found in King’s stage 1 decreases in King’s stage 2 and finally, disappears in King’s stage 3, possibly reflecting a facilitation for TDP-43 propagation to this region, which becomes affected in later phases of the disease.^4^ Nevertheless, other possible interpretations should be considered. Liu *et al*.^21^ cross-sectionally investigated *in vivo* hippocampal atrophy patterns in different King’s stages of ALS using structural MRI. They observed differential patterns of atrophy across stages 1, 2 and 3, showing early detectable hippocampal changes in stage 1. The authors suggested that ALS patients may present TDP-43-independent co-pathology, possibly due to Alzheimer’s disease pathology. This hypothesis should be taken into account in longitudinal studies combining imaging and wet biomarkers to disentangle the interpretation of our findings regarding the connectivity between the seed region and medial temporal lobes. In contrast, the connectivity with the cerebellum occurs in King’s stage 2 and decreases in King’s stage 3, and may indicate a compensatory mechanism that becomes exhausted as the disease progresses. Indeed, the hypothesis that cerebellar compensatory mechanisms are implicated in both ALS and other neurodegenerative diseases, as well as in multiple forms of neuronal damage, has been postulated, to the point of the concept of a cerebellar reserve being proposed. This term refers to the cerebellum’s unique ability to compensate for damage or loss of function caused by various conditions, with adaptive mechanisms occurring outside or within the affected area depending on the acute/focal or chronic/diffuse nature of the pathogenic event (structural and functional cerebellar reserve, respectively).^22-24^ Furthermore, clusters of relative hypermetabolism involving the cerebellum have been reported in previous ^18^F-FDG-PET studies in ALS and have been found to characterize subgroups of patients with greater survival.^25-28^

A substantial proportion of the numerous studies on connectivity in ALS^29^ employed MRI (mostly diffusion tensor imaging and functional MRI), often with small sample sizes, and only a few used ^18^F-FDG-PET/CT^14,30^ or ^18^F-FDG-PET/MRI imaging.^31^ There is mounting evidence of greater impairment to structural connectivity, primarily concentrated within the sensorimotor network. A decline in fractional anisotropy has been observed along motor and non-motor tracts in patients with ALS in comparison to control subjects, including those with lower motor neuron disease. This decline has been correlated with the progression rate in some instances.^32-37^ Conversely, functional studies have produced discordant results, for both the type and the regional pattern of connectivity changes. As compared with controls, decreased functional connectivity has been described in the premotor cortex, corpus callosum, hippocampus and cerebellum in patients with ALS.^38-43^ In contrast, other MRI studies have reported an opposite trend in largely overlapping regions, even those showing reduced structural connectivity.^44-47^ Interestingly, some MRI studies correlated higher functional connectivity with the severity of the disease, as assessed by different clinical measures (including ALSFRS-R, Medical Research Council score or cognitive function), but not by clinical stage.^48-51^ Longitudinal studies, which are often proposed as a solution to overcome these discrepancies, are strongly limited by the progressive nature of the disease. In a study including 25 patients with ALS, an increased resting state functional connectivity was observed within the fronto-striatal and the fronto-parietal networks at a 6-month follow-up.^52^ Nonetheless, only in a few studies connectivity changes were correlated with clinical stages. Spinelli *et al*.^17^ found divergent patterns of functional connectivity between King’s stages 3 and 4, with the former showing an increase in basal ganglia and temporal circuits and the latter a decrease in frontotemporal circuits.^17,29^

We acknowledge that our study has several limitations. First, the cross-sectional design of the present study still represents its main limitation. The recognized challenges associated with conducting longitudinal neuroimaging studies in a rapidly disabling disease such as ALS can lead to a high risk of attrition and consequent selection bias of subjects with slow disease progression. In this context, a proposed solution is use of the King’s clinical staging system. It has been demonstrated to serve as a reliable surrogate marker for disease progression, as the majority of patients progress from one stage to the next one, without undergoing reversions to earlier stages.^53^ Nevertheless, our interpretation of connectivity changes in terms of spread of TDP-43 proteinopathy or compensatory strategies remains speculative in the absence of longitudinal data. Second, MRI was not available for all the subjects included in this study and a future perspective is the enrolment of a cohort in which both brain ^18^F-FDG-PET and MRI data are available in order to give a more comprehensive and integrated view of metabolic, structural and functional connectivity changes occurring at different disease stages. Third, although IRCA is a consolidated method to evaluate brain metabolic connectivity, it is based on correlations among brain region metabolism and does not convey any information about directionality. Moreover, correlation is not causation. Therefore, pathophysiological hypotheses based on IRCA should be cautious. Fourth, we acknowledge that seeds derived from the same voxel-wise analysis may inflate results of the IRCA, and that using independent seeds would reduce this bias. Nevertheless, data-driven identification of seeds prevents the loss of information due to *a priori* assumptions. Had we focused solely on atlas-derived motor regions’ connectivity across King’s motor stages, we would have missed valuable information regarding the involvement of cognitive areas across different King’s stages. Indeed, data-driven identification of seeds has been extensively adopted in the published literature to investigate brain metabolic connectivity in various neurological disorders.^54-57^ Finally, although we excluded patients with major systemic illnesses potentially influencing brain metabolism, major vision disturbances, psychiatric illnesses, drug and/or alcohol addiction and other diseases potentially affecting brain functioning and metabolism, minor effects of daily pharmacological treatments on brain metabolism cannot be excluded.

To our knowledge, no other ^18^F-FDG-PET or connectivity study has included such a high number of patients with ALS. This strength led to a very robust statistical significance of the results.

In conclusion, this ^18^F-FDG-PET study investigated with high statistical power the metabolic connectivity across ALS motor stages, defined according to the King’s staging system. As King’s stage increases, we found a decrease of connectivity within the sensorimotor and cognitive areas. The larger extent of connectivity of motor and cognitive areas with temporal and cerebellar regions might reflect the spread of TDP-43 proteinopathy or a compensatory mechanism, respectively. The collection of longitudinal ^18^F-FDG-PET scans may enhance the understanding of ALS pathophysiology and allow tracking disease progression. In this context, the investigation of brain connectivity may elucidate both the trajectories of degeneration spread and the recruitment of compensatory circuits. These data might pave the way for use of the two sides of brain metabolic connectivity changes (i.e. both derangement and increased connectivity), integrated with the King’s staging system, as proxies of disease progression or coping mechanisms in clinical trials.

## Supplementary Material

awag159_Supplementary_Data

## Data Availability

The data that support the findings of this study are available on request from the corresponding author. The data are not publicly available due to privacy or ethical restrictions.
