# Supplementary material for: King’s stages of amyotrophic lateral sclerosis: an 18F-FDG-PET study of brain connectivity
Source: Brain. 2026 May 8;149(7):2495–504. doi: 10.1093/brain/awag159 (PMC13337229; doi:10.1093/brain/awag159)
Supplement: awag159_Supplementary_Data [file awag159_supplementary_data.pdf]

Supplementary methods

List of neuropsychological tests. Executive function was tested with Letter Fluency test (FAS), Category Fluency Test (CAT), Trail Making Test B-A (TMT B-A), Frontal Assessment Battery (FAB), ECAS Executive Function score, and ECAS Verbal Fluency score; Language was assessed with ECAS Language subscore and Boston Naming Test; Verbal Memory with Rey Auditory Verbal Learning Test, Immediate Recall (RAVL-IR), Rey Auditory Verbal Learning Test, delayed Recall (RAVL-DR), Babcock Story Recall Test, Immediate Recall (BSRT-IR), Babcock Story Recall Test, Delayed Recall (BSRT-DR), and ECAS Memory score; Visual Memory with Rey-Osterrieth Complex Figure Test, differed recall (ROCF-DR); Visuoconstructive abilities with Rey-Osterrieth Complex Figure Test, Immediate Recall (ROCF-IR), Clock Drawing Test (Clock), and ECAS Visuospatial Abilities score; Attention/Working memory with Digit Span Forward (FW) and Digit Span Backward (BW); Psychomotor speed with Trail Making Test A (TMT A); Non-Verbal Intelligence with Raven's Coloured Progressive Matrices (CPM47); Cognitive flexibility with Trail Making Test B (TMT B); Theory of Mind (social cognition) with Story-based Empathy Task (SET); Behaviour with the family-form of the Frontal Systems Behaviour Scale (FrSBe), ECAS, and Frontal Behavioural Inventory (FBI).

Supplementary Table 1 Results of multiple regression analysis: clusters of negative correlation between brain metabolism and King's stage. BA=Brodmann Area. Cluster extent=voxel count.

P FWE-corrected	Cluster extent	Z-score	Talairach coordinates (x, y, z)			Side	Lobe	Gyrus/ Nucleus	BA
<0.0001	12323	6.47	-14.0	-24.0	71.0	Left	Frontal	Precentral	4
		6.18	-42.0	-17.0	45.0	Left	Frontal	Postcentral	4
		5.94	46.0	-15.0	56.0	Right	Parietal	Postcentral	3
		5.84	44.0	-18.0	62.0	Right	Frontal	Precentral	4
		5.22	-32.0	-7.0	50.0	Left	Frontal	Precentral	6
		5.17	34.0	-1.0	50.0	Right	Frontal	Middle Frontal	6
		4.94	42.0	11.0	34.0	Right	Frontal	Middle Frontal	9
		4.92	12.0	-18.0	69.0	Right	Frontal	Precentral	6
		4.86	34.0	22.0	52.0	Right	Frontal	Superior Frontal	8
		4.26	-59.0	13.0	21.0	Left	Frontal	Inferior Frontal	45
		4.20	-34.0	20.0	49.0	Left	Frontal	Superior Frontal	8
0.010	727	4.85	6.0	-4.0	33.0	Right	Limbic	Cingulate	24
		3.73	10.0	15.0	38.0	Right	Frontal	Cingulate	32
		3.59	14.0	23.0	27.0	Right	Limbic	Anterior Cingulate	32
		3.43	8.0	29.0	39.0	Right	Frontal	Medial Frontal	8

Supplementary Figure 1 Scatterplot of negative correlation between brain metabolism and King's stage

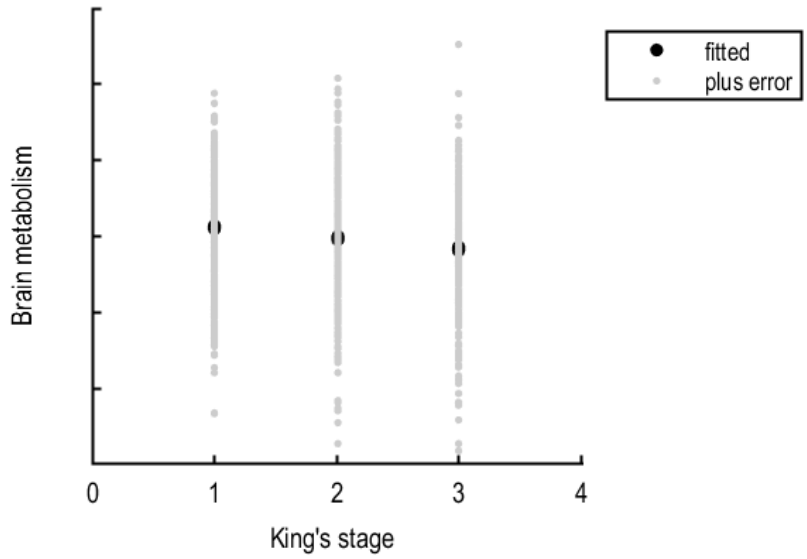

Supplementary Figure 2 Results of the sensitivity analyses (SPM12 outputs) in comparison to the original analysis

Original analysis

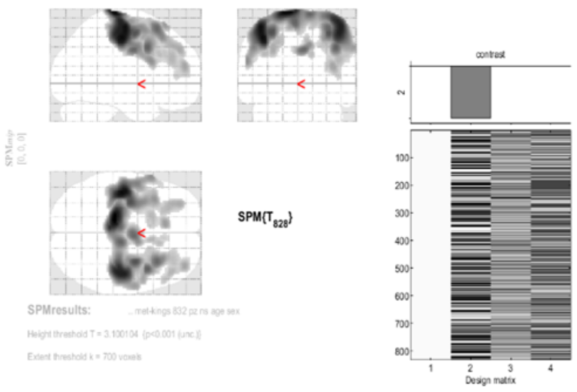

Statistics: ***p-values adjusted for search volume***

set-level p	set-level c	cluster-level $p_{FWE-corr}$	cluster-level $q_{FDR-corr}$	cluster-level k_E	cluster-level p_{uncorr}	peak-level $p_{FWE-corr}$	peak-level $q_{FDR-corr}$	peak-level T	peak-level (Z_E)	peak-level p_{uncorr}	mm mm mm
0.000	2	0.000	0.000	12323	0.000	0.000	0.000	6.56	6.47	0.000	-14 -28 76
						0.000	0.000	6.26	6.18	0.000	-42 -20 48
						0.000	0.000	6.25	6.18	0.000	-44 -16 58
		0.010	0.021	727	0.003	0.008	0.008	4.89	4.85	0.000	6 -6 36
						0.434	0.337	3.75	3.73	0.000	10 14 42
						0.589	0.457	3.61	3.59	0.000	14 22 30

Sensitivity analysis for site of onset

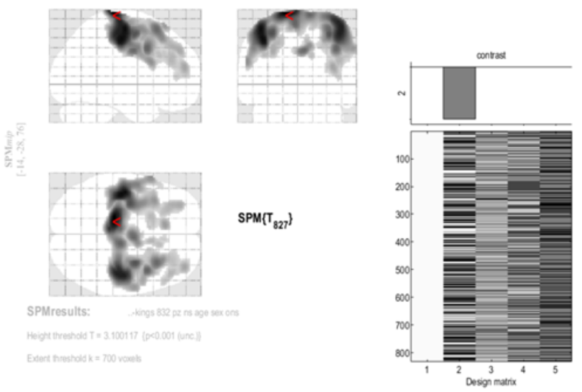

Statistics: ***p-values adjusted for search volume***

set-level p	set-level c	cluster-level $p_{FWE-corr}$	cluster-level $q_{FDR-corr}$	cluster-level k_E	cluster-level p_{uncorr}	peak-level $p_{FWE-corr}$	peak-level $q_{FDR-corr}$	peak-level T	peak-level (Z_E)	peak-level p_{uncorr}	mm mm mm
0.000	2	0.000	0.000	12308	0.000	0.000	0.000	6.55	6.47	0.000	-14 -28 76
						0.000	0.000	6.27	6.19	0.000	-42 -20 48
						0.000	0.000	6.25	6.18	0.000	-44 -16 58
		0.011	0.022	718	0.003	0.008	0.009	4.89	4.85	0.000	6 -6 36
						0.438	0.349	3.75	3.73	0.000	10 12 42
						0.593	0.469	3.60	3.59	0.000	14 22 30

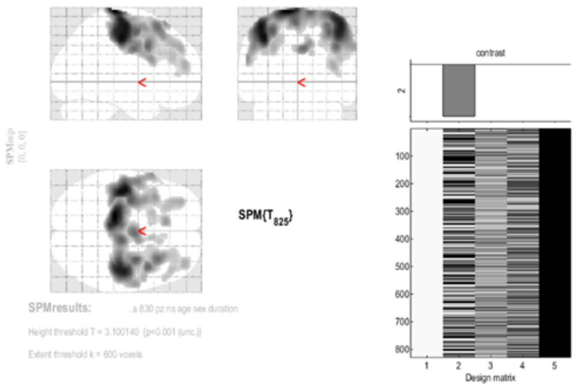

Statistics: ***p-values adjusted for search volume***

set-level p	set-level c	cluster-level $p_{FWE-corr}$	cluster-level $q_{FDR-corr}$	cluster-level k_E	cluster-level p_{uncorr}	peak-level $p_{FWE-corr}$	peak-level $q_{FDR-corr}$	peak-level T	peak-level (Z_E)	peak-level p_{uncorr}	mm mm mm
0.000	2	0.000	0.000	11709	0.000	0.000	0.000	6.58	6.49	0.000	-14 -28 76
						0.000	0.000	6.28	6.20	0.000	-42 -20 48
						0.000	0.000	6.24	6.17	0.000	-44 -16 58
		0.018	0.036	635	0.005	0.011	0.013	4.81	4.77	0.000	4 -6 34
						0.509	0.468	3.67	3.66	0.000	10 12 42
						0.699	0.585	3.50	3.49	0.000	14 22 30

Sensitivity analysis for disease duration at PET

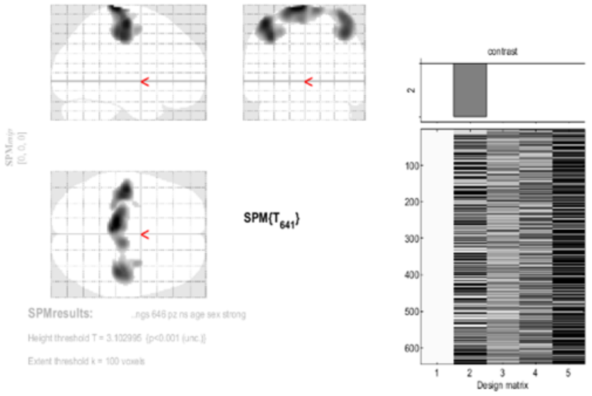

Statistics: ***p-values adjusted for search volume***

cluster-level $p_{FWE-corr}$	cluster-level $q_{FDR-corr}$	cluster-level k_E	cluster-level p_{uncorr}	peak-level $p_{FWE-corr}$	peak-level $q_{FDR-corr}$	peak-level T	peak-level (Z_E)	peak-level p_{uncorr}	mm mm mm
0.000	0.000	3494	0.000	0.000	0.001	5.65	5.58	0.000	-10 -30 76
				0.001	0.001	5.37	5.31	0.000	-46 -18 58
				0.004	0.004	5.09	5.04	0.000	-40 -22 48

Sensitivity analysis for Cognitive status at PET

Supplementary Table 2 Results of the IRCA in King's stage 1 (positive correlation). BA=Brodmann Area. Cluster extent=voxel count.

P FWE-corrected	Cluster extent	Z-score	Talairach coordinates (x, y, z)			Side	Lobe	Gyrus/ Nucleus	BA
<0.0001	44910	65535	-32.0	-3.0	52.0	Left	Frontal	Middle Frontal	6
		65535	32.0	-1.0	55.0	Right	Frontal	Middle Frontal	6
		65535	48.0	-9.0	50.0	Right	Frontal	Precentral	4
		65535	-44.0	-11.0	48.0	Left	Frontal	Precentral	4
		65535	-40.0	-19.0	43.0	Left	Parietal	Postcentral	3
		65535	-6.0	-10.0	63.0	Left	Frontal	Medial Frontal	6
		65535	8.0	-11.0	54.0	Right	Frontal	Medial Frontal	6
		65535	10.0	-7.0	63.0	Right	Frontal	Superior Frontal	6
		65535	-6.0	-13.0	43.0	Left	Frontal	Paracentral Lobule	31
		65535	-6.0	-40.0	54.0	Left	Frontal	Paracentral Lobule	5

Supplementary Table 3 Results of the IRCA in King's stage 1 (negative correlation).

BA=Brodmann Area; HC=hippocampus. Cluster extent=voxel count.

P FWE-corrected	Cluster extent	Z-score	Talairach coordinates (x, y, z)			Side	Lobe	Gyrus/ Nucleus	BA
<0.0001	631	65535	-38.0	-11.0	-16.0	Left	Temporal	Sub-Gyral	20
		65535	-38.0	-24.0	-9.0	Left	Sub-lobar	Caudate tail	
<0.0001	585	65535	40.0	-12.0	-15.0	Right	Temporal	Sub-Gyral	20
		6.81	34.0	-33.0	0.0	Right	Temporal	Caudate tail	
		6.58	28.0	-18.0	-11.0	Right	Limbic	Para-hippocampal	HC

Supplementary Table 4 Results of the IRCA in King's stage 2 (positive correlation).

BA=Brodmann Area. Cluster extent=voxel count.

P FWE-corrected	Cluster extent	Z-score	Talairach coordinates (x, y, z)			Side	Lobe	Gyrus/ Nucleus	BA
<0.0001	32918	65535	-38.0	-11.0	52.0	Left	Frontal	Precentral	4
		65535	44.0	-11.0	56.0	Right	Frontal	Precentral	4
		65535	-24.0	-12.0	61.0	Left	Frontal	Middle Frontal	6
		65535	30.0	-9.0	59.0	Right	Frontal	Middle Frontal	6
		65535	-6.0	-5.0	61.0	Left	Frontal	Medial Frontal	6
		65535	8.0	-3.0	52.0	Right	Frontal	Medial Frontal	6
		65535	10.0	-12.0	67.0	Right	Frontal	Superior Frontal	6
		65535	59.0	4.0	35.0	Right	Frontal	Precentral	6
		65535	-53.0	-1.0	28.0	Left	Frontal	Precentral	6
		65535	53.0	-38.0	50.0	Right	Parietal	Inferior Parietal Lobule	40

Supplementary Table 5 Results of the IRCA in King's stage 2 (negative correlation). BA=Brodmann Area; HC=hippocampus; AG=amygdala. Cluster extent=voxel count.

P FWE-corrected	Cluster extent	Z-score	Talairach coordinates (x, y, z)			Side	Lobe	Gyrus/ Nucleus	BA
<0.0001	802	65535	-10.0	-41.0	-40.0	Left	Posterior	Cerebellar Tonsil	*
<0.0001	136	7.11	30.0	-10.0	-13.0	Right	Limbic	Para-hippocampal	AG
		6.46	42.0	-7.0	-16.0	Right	Temporal	Sub-Gyral	20
<0.0001	114	6.69	-38.0	-1.0	-20.0	Left	Temporal	Fusiform	20
		6.62	-26.0	-12.0	-13.0	Left	Limbic	Para-hippocampal	HC
		6.37	-40.0	-14.0	-14.0	Left	Temporal	Sub-Gyral	21

Supplementary Table 6. Results of the IRCA in King's stage 3 (positive correlation). BA=Brodmann Area. Cluster extent=voxel count.

P FWE-corrected	Cluster extent	Z-score	Talairach coordinates (x, y, z)			Side	Lobe	Gyrus/ Nucleus	BA
<0.0001	23076	65535	38.0	-5.0	54.0	Right	Frontal	Precentral	6
		65535	-26.0	-11.0	58.0	Left	Frontal	Middle Frontal	6
		65535	-42.0	-15.0	54.0	Left	Frontal	Precentral	4
		65535	-30.0	-7.0	52.0	Left	Frontal	Precentral	6
		65535	-6.0	-8.0	63.0	Left	Frontal	Medial Frontal	6
		65535	44.0	-13.0	54.0	Right	Frontal	Precentral	4
		65535	6.0	-17.0	58.0	Right	Frontal	Medial Frontal	6
		65535	10.0	-7.0	63.0	Right	Frontal	Superior Frontal	6
		65535	-6.0	-6.0	41.0	Left	Limbic	Cingulate	24
		65535	-2.0	22.0	54.0	Left	Frontal	Superior Frontal	6
		65535	34.0	22.0	45.0	Right	Frontal	Middle Frontal	8
		65535	-8.0	-45.0	70.0	Left	Parietal	Postcentral	5

Supplementary Table 7 Results of the IRCA in King's stage 3 (negative correlation). BA=Brodmann Area. Cluster extent=voxel count.

P FWE-corrected	Cluster extent	Z-score	Talairach coordinates (x, y, z)			Side	Lobe	Gyrus/ Nucleus	BA
<0.0001	273	6.66	-36.0	-71.0	-47.0	Left	Posterior	Inferior Semi-Lunar Lobule	*
<0.0001	240	6.73	20.0	-43.0	-47.0	Right	Posterior	Cerebellar Tonsil	*
